# Order and Harmony

**Published:** 1884-09

**Authors:** 


### ORDER AND HARMONY.

THE Grand System of the Universe works with such harmony that there is never the slightest deviation from the natural laws that govern it. "Order is heaven's first law." The astronomer can calculate the exact position of any planet, at any given time, past, present or future. In this world nature carries out the same general plan of order and harmony. The regular alternation of the seasons; with the fruits of the earth in their time, and the cold blasts of winter, are parts of the same design. The lower orders of animal life are governed by laws that are as perfect as they are unchangeable. Man, with his free will, his selfishness, and his ambition, is the disturbing element in this Eden. With seeming forgetfulness of his noble origin, and of his destiny, he too often sacrifices health, and life itself, in the pursuit of a phantom. The ambition to rapidly acquire riches blunts his sensibilities, and in his dealings he experiences no pang in demanding "the pound of flesh."

Such men usually become victims of mental and bodily disease; and their lives close prematurely, and often before the first object of their ambition has been attained. An old proverb enjoins us to "hasten slowly." To gain money by honest industry, is most commendable. Occupation of mind and body in some honest and reasonably profitable business, is the greatest promoter of health and happiness and long life. Such a course brings us in harmony with the laws of nature and fits us for the enjoyment of life's blessings as they come to us, while disciplining us for a higher existence.

### A NEW KIND OF BURGLAR ALARM.

THE authorities at St. Giles, in Belgium, have supplied the police on night duty with cloth boots having India rubber soles. With these boots the police are so perfectly noiseless that they are at least placed on a footing of equal advantage with burglars.

